# Geriatric nutritional risk index as a potential prognostic marker for patients with resectable pancreatic cancer: a single-center, retrospective cohort study

**DOI:** 10.1038/s41598-022-18077-z

**Published:** 2022-08-11

**Authors:** Naotake Funamizu, Akimasa Sakamoto, Takeshi Utsunomiya, Mio Uraoka, Tomoyuki Nagaoka, Miku Iwata, Chihiro Ito, Kei Tamura, Katsunori Sakamoto, Kohei Ogawa, Yasutsugu Takada

**Affiliations:** Department of Hepatobiliary Pancreatic and Transplantation Surgery, Graduate School of Medicine, Ehime University, 454 Shizukawa, Toon-City, Ehime Prefecture 791-0295 Japan

**Keywords:** Cancer, Risk factors

## Abstract

In pancreatic cancer, postoperative complications (POCs) are associated with disease outcomes. The geriatric nutritional risk index (GNRI) is known to predict POCs after pancreatoduodenectomy (PD) or distal pancreatectomy (DP) in patients with hepatobiliary pancreatic tumors, including pancreatic cancer. Through POC occurrence risk, we aimed to determine whether GNRI could predict prognosis in patients who underwent PD or DP for resectable pancreatic cancer. This retrospective study examined 139 patients who underwent radical pancreatectomy for resectable pancreatic cancer at Ehime University. All patients were subjected to nutritional screening using GNRI and were followed up for POC diagnosis and patient outcomes such as overall survival (OS). Patients were divided based on the GNRI value of 99 (Low group: N = 74, GNRI < 99; High group: N = 65, GNRI ≥ 99), which was determined by receiver operating characteristic curve analysis. Multivariate analysis showed that GNRI < 99 was statistically correlated with POCs after curative pancreatic resection (p = 0.02). Univariate and multivariate analyses confirmed that GNRI < 99 was significantly associated with long OS (p = 0.04). GNRI could be a potential prognostic marker for resectable pancreatic cancer after curative pancreatic resection despite being a simple and noninvasive approach.

## Introduction

Pancreatic resection, such as pancreatoduodenectomy (PD) and distal pancreatectomy (DP), has been the gold standard surgical method for malignant pancreatic tumors. Despite the progress in surgical skills, energy devices, and perioperative management, surgery-related mortality rates following PD and DP can be up to 5%^1–3^. Among the potential postoperative complications (POCs), the most common are surgical site infections (SSIs), delayed gastric emptying (DGE), and postoperative pancreatic fistula (POPF). Particularly, SSIs—including intra-abdominal surgery-related and wound infections—and post-pancreatectomy hemorrhage (PPH) are occasionally caused by POPF^4^. Recent evidence revealed that POPF was associated with a poor prognosis in patients with pancreatic cancer^5^. The reported POCs, including the POPF rate, were 30–50%^6,7^ in patients with DP and 40% in those with PD^1^. Previously, we showed that a low geriatric nutritional risk index (GNRI) could predict SSI, POPF, and PPH in patients who underwent PD or DP for hepatobiliary diseases^8–11^. GNRI has favored assessing elderly patients’ nutritional status and predicting clinical outcomes^12^.

More importantly, GNRI is easily accessible and inexpensive, requiring only data on body weight, height, and serum albumin levels. Thus, this study evaluated the association between GNRI and outcomes such as overall survival (OS) in patients who underwent PD or DP for resectable pancreatic cancer. Identifying prognostic markers for resectable pancreatic cancer may help identify patients with a high risk of a poor prognosis.

## Materials and methods

### Patients

Between August 2013 and December 2020, 169 patients who underwent surgery for pancreatic cancer were enrolled in this study at the Department of Hepatobiliary and Transplantation Surgery, Ehime University Graduate School of Medicine, Japan. Exclusion criteria were as follows: cases of (1) exploratory laparotomy due to peritoneal dissemination, (2) choledochojejunostomy, and (3) total pancreatectomy. Moreover, (4) patients at Stage 0 and/or lacking information about the outcomes were excluded. All surgical procedures were performed by surgeons with substantial experience in pancreatic surgery. The definition of POC was defined and clarified according to the Clavien–Dindo (CD) classification^13^ and the International Study Group for Pancreatic Surgery in 2016^14^. In this study, CD grade ≥ IIIa was defined as a POC. Clinical data were collected from both inpatient and outpatient medical records. This study was approved by the ethical committee of the Ehime University in 2021 (Approval number: 2101214) and followed the Declaration of Helsinki as revised in 2013. All patients provided informed consent prior to enrollment in this study. In addition, retrospectively registered patients or their guardians, provided verbal consent to use their medical information for scientific research.

### Collection of clinical and laboratory data

As preoperative parameters, clinical data were obtained and analyzed, which included demographic variables (e.g., sex and age), anthropometric parameters (e.g., height, weight, and body mass index [BMI]), American Society of Anesthesiologist (ASA)’s physical status classification, comorbidities including diabetes mellitus, location of the tumors, neoadjuvant chemotherapy, preoperative albumin value, and GNRI. Moreover, data on intraoperative or postoperative parameters, surgery duration, estimated blood loss, blood transfusion, POC, POPF, SSI, DGE, and PPH were collected from individual medical records. The pathological stage was assigned following the TNM classification (8th edition)^15^.

### Definition of GNRI

GNRI was measured by body weight, height, and serum albumin. Data obtained before surgical procedure were as follows: GNRI = [14.89 × serum albumin (g/L)] + [41.7 × present/ideal body weight (kg)]. The ideal body weight was defined as 22 × patient height (m)^2^. If the current body weight was higher than the ideal body weight, the present/ideal body weight was 1^12^.

### Perioperative management and follow-up study

All patients preoperatively underwent routine blood tests—including serum albumin and tumor marker assessment—and a physical examination that included the measurement of body weight and height. Prophylactic antibiotics were administered through a peripheral vein before anesthetic induction. All patients who underwent DP or PD under general anesthesia received proton pump inhibitors. Amylase values from ascitic fluid obtained from the drainage tube were measured on postoperative days 1, 2, 3, 5, and 7 until removing the drains. Furthermore, dynamic computed tomography was performed to evaluate fluid collections before decannulation of the drainage tubes. Almost all patients were followed up every three months in the first two years and every six months in the following 3–5 years.

In addition, most patients undertook adjuvant chemotherapy due to S-1 for 6 months. The follow-up period started on the date of surgical procedure and ended on the date of death, at the last follow-up, or after a maximum of 60 months. OS was evaluated based on the cause of death as determined from medical records or letters and calculated using the period from the date of surgery to the date of death from any cause or last follow-up.

### Statistical analysis

All statistical significances were calculated using GraphPad Prism v5.0 (GraphPad Software Inc., La Jolla, CA, USA) and SPSS (SPSS Inc., Chicago, IL, USA). Patient backgrounds were expressed as median and interquartile ranges for nonparametric distribution. Categorical data were expressed as numbers and percentages. On the other hand, statistical significance was determined using Mann–Whitney's U test, χ^2^ test, or Fisher’s exact test for patient backgrounds and outcomes. A receiver operating characteristic (ROC) curve was analyzed to identify the optimal cutoff value of GNRI for evaluating the risk of POCs. In addition, cutoff value was determined using Youden-Index. OS following pancreatic resection was analyzed by the Kaplan–Meier method, and survival curves were compared using the log-rank test with p-values and 95% confidence intervals (CIs) of hazard ratios (HRs). Univariate and multivariate Cox proportional hazard regression models were used to identify independent prognostic factors affecting OS. A cutoff value for continuous variables was calculated by the respective median. The probability of p < 0.05 was considered statistically significant.

## Results

### GNRI and clinicopathological features

In this study, 169 patients underwent surgical procedure for pancreatic cancer in the same term. Except for 30 cases of exclusion criteria (total pancreatectomy: 3 patients, Stage 0: 3, Not pancreatic cancer: 8, No information of the prognosis: 16), 139 patients were enrolled. Patients were divided into two groups according to the presence or absence of POCs, which were CD classification^13^ ≥ grade IIIa. Table 1 summarizes clinical and demographic data from each group. No statistically significant differences in sex, age, BMI, ASA classification, presence of diabetes mellitus, neoadjuvant chemotherapy, surgical procedures, surgery duration, estimated blood loss, presence of blood transfusions, adjuvant chemotherapy (gemcitabine or S-1 for 6 months), and the completion rate, and time to adjuvant chemotherapy after surgery were observed between patients with (N = 33) and without (N = 106) POC. However, statistically significant differences were observed for preoperative albumin (p < 0.001), surgical procedure (DP or PD; p = 0.04), GNRI values (p < 0.001), and postoperative hospital stays (p < 0.001).

**Table 1:** Comparison of characteristics of patients with (+) and without (−) postoperative complications.

Characteristics	POC (+)	POC (−)	p-value
	(33)	(N = 106)	
**Sex**			
Male vs. Female	16 vs. 17	58 vs. 48	0.55
Age (years)	75 (46–87)	70 (34–89)	0.20
Body mass index	21.5 ± 0.7	22.2 ± 0.3	0.71
**ASA classification, n (%)**			
1 or 2	30 (90.9)	100 (94.3)	0.44
3	3 (9.1)	6 (5.7)	
Diabetes mellitus, n (%)	15 (45.4)	40 (37.7)	0.41
Neoadjuvant chemotherapy, n (%)	2 (6.1)	10 (9.4)	0.73
Preoperative albumin (g/L)	3.6 ± 0.1	3.9 ± 0.1	< 0.001
Operation method (DP or PD)	7/26	45/61	0.04
GNRI	91.8 ± 1.6	98.1 ± 0.7	< 0.001
Surgery duration (min)	527.4 ± 30.4	501.7 ± 15.3	0.35
Estimated blood loss (mL)	1019.0 ± 112.6	889.1 ± 77.9	0.13
Blood transfusion, n (%)	11 (33.3)	21 (19.8)	0.10
Postoperative hospital stay (days)	44.1 ± 4.1	28.8 ± 1.8	< 0.001

POC, postoperative complications; ASA, American Society of Anesthesiologists; DP, distal pancreatectomy; PD, pancreatoduodenectomy; GNRI, geriatric nutritional risk index.

### Optimal GNRI cutoff value calculation by ROC curve

**Figure 1:**
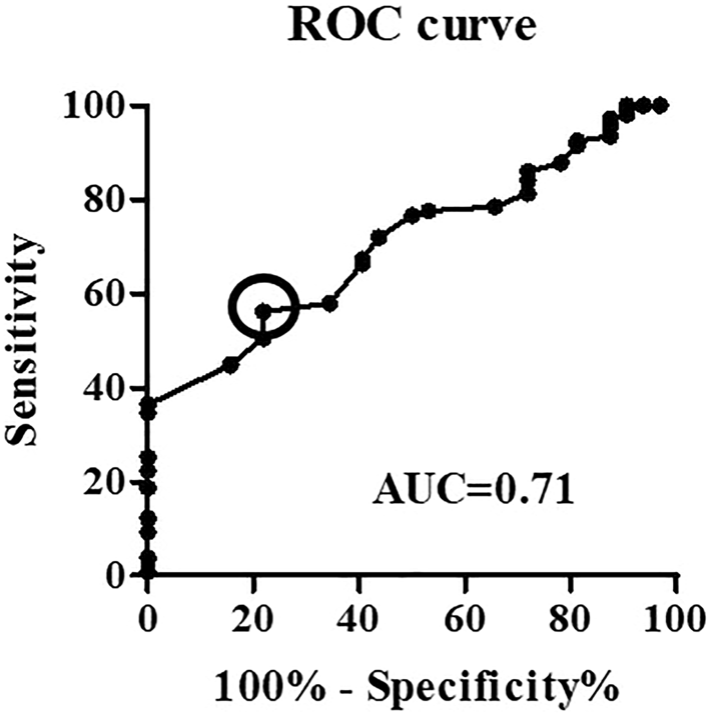
Selection of GNRI cutoff value using receiver operating characteristic (ROC) curve analysis.

The optimal cutoff value for evaluating the risk of POCs was determined using ROC curve analysis (Fig. 1). With an area under the curve of 0.71 (95% CI: 0.62–0.80), the most appropriate cutoff value was determined to be 99. This value had a sensitivity of 56.6% and a specificity of 78.8%. Patients were then divided into two groups according to the established cutoff value: Low (GNRI < 99, N = 72) and High (GNRI ≥ 99, N = 67) groups. The background profiles were compared between these two groups (Table 2). BMI, preoperative albumin, surgical procedure, and POC rate varied significantly between the two groups. The observed POC rates and the detail in the Low and High groups were 34.7% (N = 25) and 10.4% (N = 7), respectively (Table 3). Univariate analysis revealed that the Low group had a significantly higher rate of POC incidence than the High group (Table 3; p = 0.001).

**Table 2:** Comparison of patient characteristics between the study groups.

Characteristics	Low	High	p-value
	(N = 72)	(N = 67)	
Sex (male vs. female)	37 vs. 45	29 vs. 40	0.74
Age (years)	72.5 (46–87)	70 (34–89)	0.14
Body mass index	21.1 ± 0.4	23.5 ± 0.4	< 0.001
**ASA classification, n (%)**			
1 or 2	68 (94.4)	62 (92.5)	0.74
3	4 (5.6)	5 (7.5)	
Diabetes mellitus, n (%)	30 (41.7)	25 (37.3)	0.61
Neoadjuvant chemotherapy (%)	7 (9.7)	5 (7.5)	0.77
Preoperative albumin (g/L)	3.5 ± 0.1	4.2 ± 0.1	< 0.001
DP or PD	21/51	35/32	0.04
Operation time (min)	529.6 ± 20.2	483.9 ± 17.9	0.11
Estimated blood loss (mL)	889.4 ± 95.1	950.9 ± 89.6	0.65
POCs (CD grade over IIIa, %)	26 (36.1)	7 (10.4)	0.001
Stage I, II/III, IV	64/8	61/6	0.78
Postoperative hospital stays (days)	34.9 ± 2.9	29.6 ± 1.8	0.11
AC Induction (%)	56 (77.8)	54 (80.6)	0.68
AC Completion (%)	29 (51.8)	34 (63.0)	0.23
Time to AC after surgery (days)	54.5 ± 5.7	42.5 ± 3.3	0.07

ASA, American Society of Anesthesiologists; DP, distal pancreatectomy; PD, pancreatoduodenectomy; POCs, Postoperative complications; AC, adjuvant chemotherapy.

**Table 3:** Comparison of the frequency of postoperative complications (CD grade≧III) between the study groups.

Variables	Low	High	p-value
	(N = 72)	(N = 67)	
All POCs (CD grade over IIIa, %)	26 (36.1)	7 (10.4)	0.001
Bile leakage, n (%)	1 (1.4)	3 (4.5)	0.35
POPF, n (%)	9 (12.5)	3 (4.5)	0.13
PPH, n (%)	5 (6.9)	0 (0)	0.06
Chylorrhea, n (%)	1 (1.4)	0 (0)	0.33
SSI, n (%)	6 (8.3)	0 (0)	0.03

POCs, postoperative complications; POPF, postoperative pancreatic fistula; PPH, postpancreatectomy hemorrhage; SSI, surgical site infections; pancreatoduodenectomy; CD, Clavien–Dindo Classification.

### OS due to the cutoff value of GNRI

**Figure 2:**
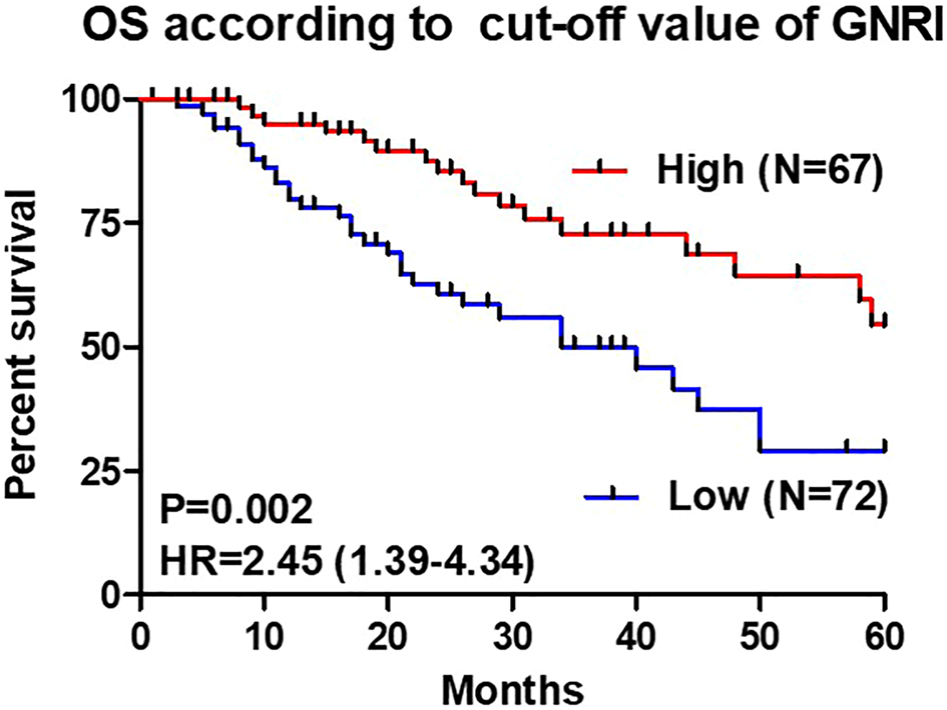
Overall survival curves between the Low and High GNRI groups. The High group shows a better prognosis than the Low group.

**Figure 3:**
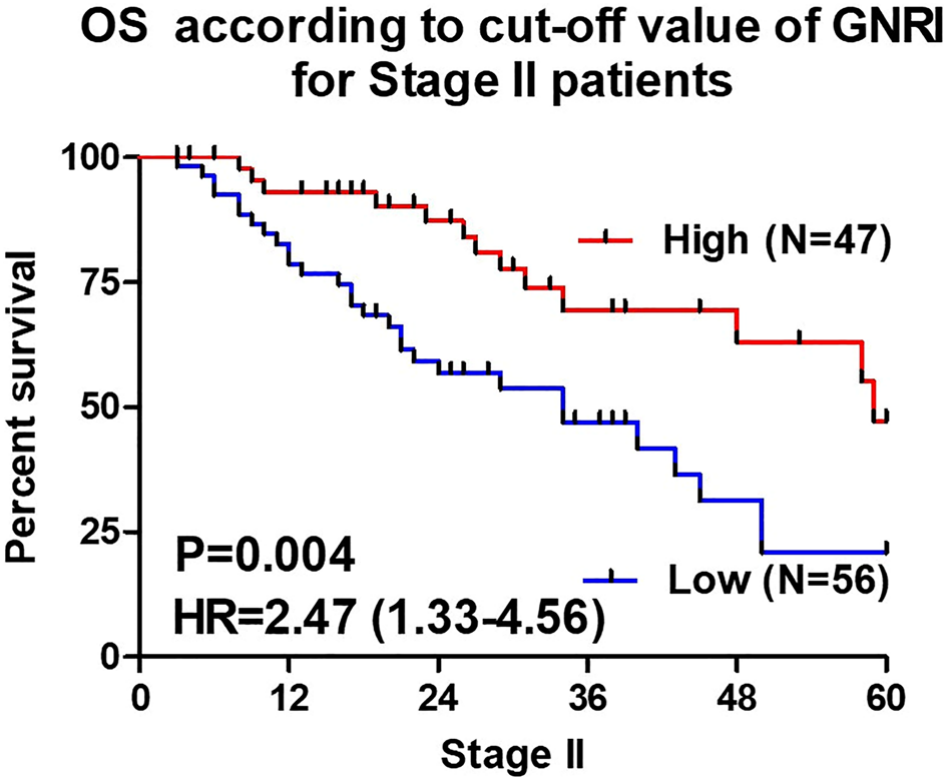
Overall survival curves between the Low and High GNRI groups in Stage II patients. OS is associated with a low GNRI level in Stage II patients.

Kaplan–Meier analysis and the log-rank test demonstrated that patients in the Low group had a significantly worse prognosis in terms of OS than those in the High group (p = 0.002; Fig. 2). GNRI value was significantly associated with OS as a prognostic marker. Moreover, Stage II patients were compared according to GNRI cutoff value because they accounted for 77.8% and 70.1% of the Low and High groups, respectively (Fig. 3).

### Cox regression analysis for OS

Univariate analysis showed that OS was significantly associated with GNRI cutoff value (p = 0.003). Multivariate analysis revealed that GNRI < 99 (HR: 2.45; 95% CI: 1.02–5.86; p = 0.04), sex (HR: 3.01; 95% CI: 1.57–5.77; p = 0.001), and surgical procedure (HR: 2.30; 95% CI: 1.16–4.55; p = 0.02) were significant independent prognostic potential markers for OS (Table 4).

**Table 4:** Prognostic factors for the overall survival in patients with pancreatic cancer.

Characteristics	Univariate analysis			Multivariate analysis		
	HR	95% CI	p-value	HR	95% CI	p-value
Sex (Male vs. Female)	2.82	1.50–5.33	0.001	3.14	1.66–5.95	0.001
Age (> 70 years)	0.96	0.55–1.69	0.90			
Body mass index (< 22)	1.01	0.91–1.10	0.98			
ASA classification 1 or 2 vs. 3	2.36	0.92–6.01	0.07			
Preoperative albumin (< 3.6 g/L)	0.56	0.31–1.00	0.050			
CEA (> 3.6 ng/mL)	1.66	0.92–2.99	0.09			
CA19-9 (> 52.0 U/mL)	1.34	0.76–2.35	0.31			
GNRI (< 99)	2.46	1.36–4.44	0.003	2.49	1.37–4.54	0.003
Operation methods (PD or DP)	2.58	1.32–5.01	0.005	2.32	1.17–4.61	0.02
Postoperative complications	1.62	0.90–2.92	0.11			
Stage I, II/ III, IV	1.02	0.40–2.56	0.97			
Adjuvnt chemotherapy	1.12	0.65–2.84	0.70			

ASA, American Society of Anesthesiologists; GNRI, geriatric nutritional risk index; PD, pancreatoduodenectomy; DP, distal pancreatectomy.

## Discussion

Despite the advanced surgical procedure and perioperative management, POCs after PD or DP (rate: 40–60%) remain a cause of high morbidity in patients with pancreatic cancer^16,17^. The most common major POCs are the development of POPF, DGE, intra-abdominal infection, and PPH. POPF is particularly known as major POC, which is a potentially fatal complication after PD or DP^16,17^. Several studies have reported that POPF was associated with sex, BMI, blood transfusion, pancreatic texture, preoperative biliary drainage, lower serum albumin level, CRP level, and nutritional status^3,18^. However, a definitive risk factor for POPF remains incompletely understood^19^. Generally, POCs have been associated with poor OS, likely due to chemotherapy delay and cancer progression caused by chemokines/cytokines induced POC-associated inflammation in several type of cancers including pancreatic cancer^5,20,21^.

Therefore, the primary goal of the surgeons is to prevent these POCs, especially POPF, to reduce poor survival. Previous studies have also reported pancreatic volume, completion of adjuvant chemotherapy, cancer antigen 19-9, carcinoembryonic antigen level, and prognostic index as prognostic factors in patients with pancreatic cancer^22–24^.

Recent evidences revealed that nutritional status such as prgnostic nutritional index (PNI) and GNRI are strongly associated with POCs or patients outcomes^25,26^. The PNI is defined using serum albumin and lymphocyte count. Luan et al.^25^ showed that levated PNI is correlated with a better prognosis in head and neck patients. However, AUC was compared with PNI and GNRI using ROC curve analysis. As the result, AUC showed that GNRI was higher than PNI (GNRI: 0.71 *vs* PNI: 0.60) in this study. On thie other hand, Hayama, et al.^26^ reported that lower GNRI was significantly associated with a poor prognosis compared to PNI in elderly patients with colorectal cancer. Thus, we believe GNRI will be better prpgnostic marker than PNI.

Using the nutritional status as an objective nutritional screening tool, Bouillanne et al.^12^ first reported that GNRI, which includes albumin and BMI, is a prognostic factor of morbidity and mortality in hospitalized elderly patients. Subsequently, several studies have consistently shown that a relationship exists among GNRI, POCs, and cancer prognosis as well since nutritional status was strongly associated with cancer prognosis^25,27–29^. For example, Kushiyama et al.^30^ reported that GNRI is associated with POCs after gastrectomy. Furthermore, recent reports revealed that GNRI is an important predictor of POCs and OS in patients with gastric cancer^31,32^. Recent evidence revealed that GNRI is a significant prognostic factor in advanced lung^33^ and colorectal^34^ cancers. Hu et al., showed that GNRI could be a useful prognostic indicator in patients who underwent surgery^35,36^, notably, in pancreatic cancer.

Previous studies have shown that GNRI is associated with a high risk of POCs, including SSIs, POPF, and PPH after PD or DP^8–11^. Briefly, a lower GNRI value was related to a higher risk of SSI and reported to be a potential marker for developing POPF and PPH after pancreatic surgery. We have recently reported the role of GNRI as a risk factor for POPFs after DP in 37 patients with pancreatic tumor or invasive gastric cancer^8^ and for SSIs after PD in 93 patients with hepatobiliary pancreatic or duodenal cancer^11^. In the present study, we further investigated the predictive value of GNRI for not only the POCs but also long-term postoperative survival after PD or DP in 139 patients with pancreatic cancer treated in a different institution.

In this study, there were 32 patients (23.0%) who developed POCs, including POPF (10.8%), bile leakage (2.9%), PPH (3.6%), and SSIs (7.9%), after PD or DP, which corresponded to CD classification ≥ IIIa and sometimes overlapped. The GNRI value of < 99 was strongly associated with a high risk of POCs, supporting the use of nutritional assessment before an elective procedure. Furthermore, a low GNRI value was significantly associated with poor long-term prognosis. The occurrence of POCs also showed a tendency to deteriorate the OS, although not statistically significant. The development of POCs is considered a potential predictor of worse outcomes because POCs are intimately associated with the delayed induction of adjuvant chemotherapy. In addition, a preoperative poor nutritional condition reflected by a lower GNRI value can affect the postoperative recovery, tolerance for adjuvant chemotherapy, and antitumor immune defense. The exact mechanisms underlying the association between lower GNRI and poorer survival outcome should be determined in further studies. However, some reports showed that body weight or nutritional status were associated with worse prognosis in pancreatic cancer patients, which evidence strongly supported our result^34,35^. At present, our results are consistent with those of a previous study on several cancer^36–38^. Moreover, their cutoff values were close to present study, although one of them was age restricted study^39^. Thus, present study strengthened that GNRI might be useful predictor for prognosis in patients with pancreatic cancer who underwent radical surgery.

Our study has several limitations in terms of the interpretation of the study results. First, there was a lack of statistical power caused by the relatively small sample size. Second, our data were collected at a single center. The third limitation lies in the retrospective nature of the study. Finally, present study cannot break the racial line, because only asian data have been reprted. Therefore, a more comprehensive large-scale prospective study should be conducted in the future to validate our study findings.

Finally, we believe that although GNRI can be easily acquired from preoperative routine work without an invasive procedure, it can predict OS in patients with pancreatic cancer after pancreatic resection. Therefore, future prospective randomized studies are warranted to investigate the significance of GNRI for improving outcomes in patients with pancreatic cancer after curative surgery.

## Data Availability

The data is available from the corresponding author on reasonable request.
